# Effective BCDNet-based breast cancer classification model using hybrid deep learning with VGG16-based optimal feature extraction

**DOI:** 10.1186/s12880-024-01538-4

**Published:** 2025-01-08

**Authors:** Meenakshi Devi P., Muna A., Yasser Ali, Sumanth V.

**Affiliations:** 1 Department of Information Technology, K.S.R. College of Engineering, Tiruchengode, Tamilnadu 637215 India; 2 Centre for Research Impact & Outcome, Chitkara University Institute of Engineering and Technology, Chitkara University, Rajpura, Punjab 140401 India; 3 Chitkara Centre for Research and Development, Chitkara University, Baddi, Himachal Pradesh 174103 India; 4 Department of Information Technology, Manipal Institute of Technology Bengaluru, Manipal Academy of Higher Education, Manipal, Karnataka 576104 India

**Keywords:** Breast cancer diagnosis model, Ultrasound images, Breast cancer diagnosis network, Atrous spatial pyramid pooling, Random parameterized adaptive opposition slime mould with egret swarm optimization

## Abstract

**Problem:**

Breast cancer is a leading cause of death among women, and early detection is crucial for improving survival rates. The manual breast cancer diagnosis utilizes more time and is subjective. Also, the previous CAD models mostly depend on manmade visual details that are complex to generalize across ultrasound images utilizing distinct techniques. Distinct imaging tools have been utilized in previous works such as mammography and MRI. However, these imaging tools are costly and less portable than ultrasound imaging. Also, ultrasound imaging is a non-invasive method commonly used for breast cancer screening. Hence, the paper presents a novel deep learning model, BCDNet, for classifying breast tumors as benign or malignant using ultrasound images.

**Aim:**

The primary aim of the study is to design an effective breast cancer diagnosis model that can accurately classify tumors in their early stages, thus reducing mortality rates. The model aims to optimize the weight and parameters using the RPAOSM-ESO algorithm to enhance accuracy and minimize false negative rates.

**Methods:**

The BCDNet model utilizes transfer learning from a pre-trained VGG16 network for feature extraction and employs an AHDNAM classification approach, which includes ASPP, DTCN, 1DCNN, and an attention mechanism. The RPAOSM-ESO algorithm is used to fine-tune the weights and parameters.

**Results:**

The RPAOSM-ESO-BCDNet-based breast cancer diagnosis model provided 94.5 accuracy rates. This value is relatively higher than the previous models such as DTCN (88.2), 1DCNN (89.6), MobileNet (91.3), and ASPP-DTC-1DCNN-AM (93.8). Hence, it is guaranteed that the designed RPAOSM-ESO-BCDNet produces relatively accurate solutions for the classification than the previous models.

**Conclusion:**

The BCDNet model, with its sophisticated feature extraction and classification techniques optimized by the RPAOSM-ESO algorithm, shows promise in accurately classifying breast tumors using ultrasound images. The study suggests that the model could be a valuable tool in the early detection of breast cancer, potentially saving lives and reducing the burden on healthcare systems.

## Introduction

One of the most hazardous diseases to impact people is cancer. It is a condition in the cells, which are the smallest unit of the human body, proliferate and inhibit improperly, destroying healthy tissues [1]. Breast cancer is a prevalent, frequently fatal condition that causes death in women. Early detection is essential for the effective treatment of breast cancer and a reduction in mortality since the origins of breast cancer remain unclear [2]. Ultrasound imaging has been utilized for screening and detecting breast diseases because of its noninvasiveness, mobility, low cost, and real-time imaging capabilities [3]. Most of the research showed that ultrasonography should reliably identify both benign and malignant tumors.An ultrasonic technique is called elastography and it is used to assess tissue hardness [3]. Elastography is useful in distinguishing between benign and malignant breast lesions since there is a clear difference in elasticity between benign tumors and malignant [3]. Breast tumors are identified by shear wave elastography and strain elastography. Strain elastography is mostly used for breast diagnosis to provide elastic images [4]. The determined stain elastography tissue is compared to other tissues and it easily validates the tissue hardness.

Additionally, due to problems of low inter-class and high intra-class similarity, an automatic multi-class classification with ultrasound images has several difficulties [5]. The main difficulty in providing robustness for a particular activity is gathering sufficient data for model validation and training [6]. To estimate the generalizability of the trained model in complex situations, a representative independent test set is requested during the training process [7]. Particularly, for understanding the lesion variances, the training set must include a large set of data with abnormal classes thus increasing the robustness of the process [8]. This makes information collection even more difficult for tasks like screening, which is used to identify abnormal behavior. It is expensive to gather data in medical imaging with physician annotation or biopsy truth, and deep learning engineers do not have access to such resources [9]. Disease and clinical information from medical reports and pathological reports are extracted using Natural Language Processing (NLP) and information mining of the health data. The methods employed to determine the accurate extracted labels and the automatically mined disease labels [10]. The labels contain a particular amount of noise and frequently lack image or lesion-level annotations [11]. The segmentation of an ultrasonic image is significantly influenced by the image quality. The ROI and the backdrop have identical textures, which makes it very challenging to automatically detect the ROI.

The Convolutional Neural Network (CNN) has successfully been used in numerous image identification applications [12]. It has demonstrated exceptional performance in computer vision tasks. CNNs attained a lot of interest from scientists working in the medical sector [13]. However, it is exceedingly challenging to gather a significant number of labelled medical images [13] and train a model using random weight initialization. Many low-level visual characteristics are used in the network such as edges, textures, geometry, area, lighting variation, etc., for the detection of ultrasound breast cancer images [14]. Therefore, transfer learning in deep learning and CPU vision can help to solve the limited sample size issue. Hence a new breast cancer detection method is implemented related toa deep learning algorithm.

## Research motivation

The ultrasound image-based classification process of breast cancer is more cost-effective and radiation-free than conventional imaging tools. However, the ultrasound image-based classification process also causes some issues such as high similarity among the malignant and benign tumors, inaccurate classifications, and irregular tumor shapes. Therefore, to prevent these issues, the BCDNet is implemented that recognize the necessary features from the ultrasound images that help to differentiate the benign and malignant tumors and also the irregular tumor shapes accurately. Moreover, by utilizing the hybrid optimization, the BCDNet helps to improve the model efficiency and accuracy rates. For classifying breast cancers, numerous efforts have been made in the healthcare sector. The early recognition of this disease can prevent mortality rates [15]. However, the manual analysis of this disease demands more time and also effort. The process can be subjective and prone to error. Also, this manual analysis highly relies on experienced medical analysts. Therefore, developing an automated model is important. Machine learning and especially the deep learning models provide more attention to the disease classification domains since these models can provide highly desired solutions without any manual analysis. Deep learning models such as CNN can select the features automatically for the input images. However, the features extracted from the original images can also generate irrelevant features. This can affect the classification process [16]. Hence, it is significant to choose only the highly related features for precise classification. The selection of related features from the extracted features is one of the complex tasks. Numerous works utilize the selection algorithms such as PSO and GA. By utilizing these techniques, the best features are selected. But, sometimes during the feature selection based on these algorithms may lose some necessary features and also these algorithms fall into the trap of local optima. Therefore, the developed model employed transfer learning of optimized VGG 16. This technique effectively selects the features with less computational time, where also the developed hybrid algorithm named RPAOSM-ESO is utilized for weight optimization. The hybrid algorithm escapes from the existing issues in the algorithm utilizing an adaptive strategy and the VGG 16 model can capture the related features with its deep layers. Further, for the classification process, the previous machine learning models are utilized in the existing works. However, these models relied on medical expertise and provided inaccurate solutions. Therefore, deep learning-based classification is concentrated nowadays for breast cancer [17]. Although the existing models are effective in the classification tasks, these models utilize more computational resources and parameters. Moreover, the existing models can't focus on the rich features for the classification. Therefore, this work introduced a hybrid network named AHDNAM, which includes DTCN, 1DCNN, ASPP, and the attention method. Here, the ASPP is incorporated with the DTCN, and the attention method is incorporated with the 1DCNN. These two models generate the predicted scores and then, the predicted scores are averaged for achieving the classified outcome. This network effectively minimizes the limitations in the existing models and also optimally chooses the hyperparameters by the same hybrid algorithm named RPAOSM-ESO. Thus, the accuracy of the classification is maximized for breast cancer than the conventional models.

### Contributions of the developed model

- To design a breast cancer diagnosis model by utilizing ultrasound images and hybrid deep models for effectively classifying the benign or malignant tumor in the early stage. This automated model minimizes the false positives and helps to offer better treatment recommendations. Also, the model reduces manual interventions and is utilized to decrease the death rates.
- To implement an RPAOSM-ESO algorithmby integrating two algorithms such as AOSMA and ESOA with an adaptive mechanism for optimizing the weight and parameters in feature extraction and cancer classification phases respectively. This process helps to maximize the MCC and accuracy. Also, it minimizes the FNR in classification thus increasing the performance.
- To extract the significant features from the input ultrasound images by employing an effective transfer learning of VGG-16 that helps to minimize the training time and increase the model efficiency. Also, it is highly supported in the model classification process for performing accurate classifications with less amount of time. Here, the model weights are tuned by the RPAOSM-ESO algorithm for increasing the feature retrieval process’s efficacy.
- To design an AHDNAM classification model by incorporating powerful models such as 1DCNN, DTCN, and ASPP with attention method for accurately classifying the benign or malignant tumor with a high accuracy rate. In this network, the ASPP is incorporated with the DTCN model, while the attention method is incorporated to produce better predictions. The predicted scores from these models are involved with the average calculation for achieving desired solutions. Here also the RPAOSM-ESO is considered for parameter optimization.

### Organization of the developed work

The implemented breast cancer detection method utilizing hybrid deep learning contains the below sections. The Literature survey provides a brief explanation of the previously used breast cancer diagnosis models with their features and disadvantages. The architectural view of implemented breast cancer diagnosis model describes the novel strategies used in the developed model for improving breast cancer detection. The overall process in BCDNet model using deep networks with the heuristic-based optimal tuning of parameterspresents a detailed description of the developed hybrid deep learning model. The results and discussions present the experimental setup and metrics used to evaluate the model's performance. The conclusion summarizes the overall work with the key findings of the breast cancer detection model.

## Literature survey

### Related works

In 2020, Liao et al. [18] have recommended a novel tumor detection system for detecting tumors such as benign or malignant with ultrasound imaging using deep learning technology. The advanced region detection method was used to separate the lesion patches from the collected images. The detectedimages used a VGG-19 network to identify whether the breast tumor was malignant or benign. The results of the experiments demonstrated that the suggested strategy’s accuracy was nearly equal to manual segmentation. The suggested diagnostic method was experimented with other conventional methods and it achieved better performance.

In 2020, Kim et al. [19] have developed a new deep learning-based model for effectively evaluating breast cancer medical imaging. The details were extracted from an input image. Many different types of extracted line segments were identified. The input for the improved model was the compressed image. The effectiveness of the suggested approach was assessed using conventional deep-learning models. According to the findings, the suggested model had low loss and greater accuracy.

In 2020, Zeimarani et al. [20] have suggested an advanced model for identifying breast lesions from ultrasound images. In addition, this work modified severalpreviously trained methods for their data. The dataset contained 641 patient details. The classifier's results provided generalized outcomes. The image regularization and augmentation methods were used to increase the accuracy.The acquired findings showed that the implemented architecture was more successful than certain conventional learning algorithms in classifying tumors.

In 2022, Iqbal et al. [21] have implemented an attention network to simultaneously segment breast lesions images. The new block was implemented to address the traditional difficulties, extract more semantic features, and increase feature diversity. An integration of lesion attention blocks and channel-related attention, known as dual attention, was also presented. Finally, the developed models were able to focus on significant features. Two datasets and two private datasets were used for the experimental analysis.

In 2020, Aleksandar et al*.* [22] have proposed an effective breast tumor segmentation with ultrasound images to predict breast cancer. The suggested method extracted the feature areas with levels by adding attention blocks to a U-Net structure. The validation results showed improved tumor segmentation accuracy for the developed system. On a set of 510 images, the method produced a better performance. The salient attention approach offered high accuracy.

In 2022, Zhai et al. [23] have discovered a new novelmodel that usedtwo generators. The generates could build trustworthy segmentation prediction masks without labels. Therefore, model training was effectively promoted by using unlabeled cases. The three datasets were used to validate that model. The outcomes of the model demonstrated that the developed model performed better. The suggested method gave better results than fully supervised methods.

In 2022, Podda et al. [24] have designed a diagnostic method for segmenting breast cancer. That method was employed to increase the diagnosis precision rate and decrease the workload of the operator. The current study used to develop a completely automated method for the classification and segmentation. The suggested system was compared to other models like CNN architectures. Finally, this work introduced an iterative algorithm that utilized the outputs of the detection stage to enhance the detectionresult. The effectiveness of the deep approaches was demonstrated by experimental results.

In 2023, Meng et al. [25] have implemented a deep-learning method for predicting breast lesions. The designed model was used to increase the extracted feature’s performance.The attention module was employed to reduce the noise of the input image. The weights were effectively calculated using entire global channel attention modules. The public and private datasets were employed for the validation. The designed framework performance showed a better outcome. The suggested model achieved high precision among traditional models.

### Research gaps

Distinct research has been made in the current days for classifying breast cancer since it increases the death rates if it is not handled properly. Most of the time, the misclassifications occur in the manual analysis due to similar shapes and sizes of the tumors. Moreover, this manual analysis is highly expensive and consumes more time. The operator workload is also high in existing classification models. Additionally, most breast cancer classification models do not support early detection and real-time monitoring. Therefore, automated classification models attained much attention nowadays. Machine learning and deep learning models become very famous in the medical sector since these models have the ability to classify tumors more automatically and more effectively than manual analysis. Nevertheless, these model-based classification procedures also require enhancements. Several important problems of these previous works are listed below.

- ❖ The previous automatic classification models for breast cancer utilized imaging tools such as MRI, mammography, and so on. However, these imaging tools are extremely expensive and have radiation exposure. Over diagnosis is also caused by these imaging tools. Therefore, a non-invasive and low-cost imaging tool such as ultrasound images is required for the classification process.
- ❖ Feature extraction is one of the important processes in any disease classification task. However, the majority of the traditional classification works didn't consider feature extraction, which degraded the model performance and also accuracy rates. Therefore, performing a feature extraction is significant for accurate cancer classification.
- ❖ Some machine learning-assisted models utilized manual intervention for obtaining significant features. That increased the error rates and increased the overfitting. Therefore, utilizing deep learning-based approaches for extracting the features is necessary. Although some existing works employed deep learning for feature extraction, the models utilize more training time. So, including transfer learning improves the feature extraction process.
- ❖ Most of the works utilize deep learning frameworks for cancer classification nowadays since these provide the desired solutions. However, these models also struggle to provide better solutions when processing large-scale datasets or giving poor performances when the image quality is low. Therefore, developing a hybrid deep network with additional significant techniques such as attention is important for preventing these issues.
- ❖ Though some works consider the hybrid deep networks for the cancer classifications, these networks utilize more hyper-parameters that reduce the model stability and also increase the dimensionality issues. Hence, fine-tuning these model hyperparameters is relatively important.
- ❖ The existing models [26] and [27] have utilized the dual transfer learning model for resolving the issue of classified medical image inefficiency. This model minimized the requirement fora large number of classified images and also resolved the issue of field convergence. However, this model didn't perform the fine-tuning process degraded the efficiency, and also utilized more computational time.
- ❖ Traditional model [28] has analyzed distinct AI models and its complexities and features. It illustrated the power of AI mechanisms in the analysis of omics data and recognized the severe issues that should be resolved for attaining better solutions. The machine learning and deep learning models were explained for the classification tasks. However, selecting a suitable learning model for classifying diseases is always a difficult process.

These disadvantages motivate us to implement effective breast cancer segmentation and classification systems with deep learning approaches and some of the traditional model's merits and disadvantages are listed in Table 1.

**Table 1:** Advantages and challenges of breast cancer classification using deep learning

Author [citation]	Methodology	Advantages	Challenges
Liao et al*.* [18]	VGG-19	• It improves the survival rates • It gives higher segmentation accuracy	• It utilizes high cost for the implementation • It gives poor results in the presence oflow contrast and inherent noisy images
Kim et al*.* [19]	CRNN	• It effectively classifies breast cancer in the early stages • It easily classifies breast cancer in real-time imaging	• It takes more time to train the classifier • It improves the loss rates
Zeimarani et al*.* [20]	CNN	• It gives highly effective outcomes when using large-scale image datasets • It improves classification accuracy	• It struggles with overfitting problems during the training process • It causes vascular disease and skin ailments
Iqbal et al*.* [21]	MDA-Net	• It reduces the misdiagnosis issue by medical doctors • It decreases the computational burdens	• It struggles to classify cancer because of its complex shape, unclear boundary, curvature, and intensity • The classification results only depend on the quality of saliency maps
Aleksandar et al*.* [22]	U-Net	• It enhances robustness and accuracy while processing medical images • It is a safe and cheapest model	• It requires more number of parameters
Zhai et al*.* [23]	ASSGAN	• It effectively segments the breast tissue • It gives accurate breast lesion segmentation	• Due to the large variability of boundaries and shapes, it struggles to detect cancer • It decreases the sensitivity of the classification model
Podda et al*.* [24]	CNN	• It reduces the mortality rates and operator workload • It easily predicts complex and small measurements	• Its computational time is relatively high • It suffers from misclassification issues
Meng et al*.* [25]	DGANet	• It gives good generalizations • It reduces serious complications and deaths	• It does not support early-stage breast cancer detection • It is hard to detect cancer with the complex structure of breast tissue

### Architectural view of implemented breast cancer diagnosis model

**Fig. 1:**
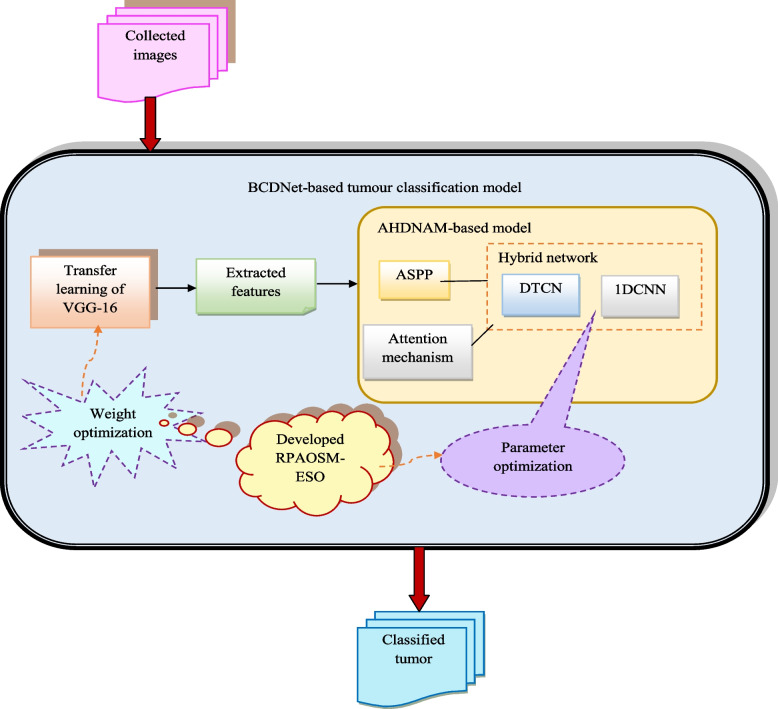
Structural depiction of the developed breast cancer detection system using hybrid deep learning

The newly designed breast cancer diagnosis model is utilized to accurately predict whether the tumor is benign or malignant. It is used to decrease the death rates. The ultrasound images are collected from online sources for predicting breast cancer. The collected ultrasound images are fed into the BCDNet-based classification model for diagnosing breast cancer. Here, feature extraction and classification are performed in BCDNet. The feature extraction is performed using transfer learning of the VGG-16 method. Here, the developed RPAOSM-ESO is used to optimize the weights to improve the classification performance. Then, the deep features are fed into the classification of the breast cancer phase. The classification uses an ASPP-based hybrid of DTCN and 1DCNN network with AM. Here, the developed RPAOSM-ESO is used for optimizing the parameters such as epochs and hidden neuron count to maximize accuracy and MCC. Also, it is effectively minimizing the FNR value. Parameter optimization is employed to enhance the effectiveness of the offered system and it accurately classifies the benign or malignant tumor. Finally, the developed breast cancer diagnosis model is compared to other models with performance measures that give increased accuracy than other models.The structural representation of the offered breast cancer diagnosis system is shown in Fig. 1.

### Ultrasound image collection

#### Dataset (CBIS-DDSM)

The ultrasound images are gathered from the online database using the link “https://www.kaggle.com/datasets/aryashah2k/breast-ultrasound-images-dataset: Access date: 2023-06-07”. It contained ultrasound images in a PNG format. Totally 103 columns are presented here. In addition, six files are presented here. Normal, benign, and malignant cases are also included in the dataset. It is a standard dataset. Totally 1578 images are here. The patient ID details are given in one column. It generally helps recent researchers who have an interest in detecting cancer disease.

**Fig. 2:**
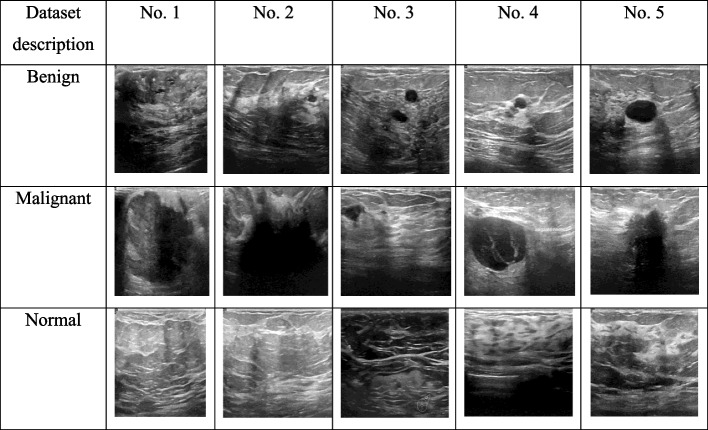
Gathered ultrasound images from online resources

Hence, the collected inputs of ultrasound images are represented by $B_{u}^{Ig}$. The total images are noted by $U$. The collected sample images from the dataset are depicted in Fig. 2.

### BCDNet: breast cancer diagnosis model with deep feature extraction

#### Transfer learning of VGG16-based breast feature extraction

The feature retrieval is performed using transfer learning of the VGG-16 method. In the feature extraction, the gathered ultrasound images are considered as an input, and it is denoted by $B_{u}^{Ig}$.

#### Transfer learning of VGG-16 [29]

Transfer learning is a machine learning mechanism. The transfer learning uses a trained CNN's feature learning layers to categorize a different problem. In other words, this technique utilizes the knowledge attained from one task to increase the performance of the related task. Here, the transfer learning of VGG 16 is utilized. Hidden layers, input, and output, are used in the VGG-16. It is one of the topmost CNN-related algorithms. This model showed an important improvement over the state-of-the-art.

When comparing with the other pre-trained models, mostly the models fail to produce the proper features also these models given redundant features. This minimized the performance of the classification process. The VGG 16 technique has 16 layers, offering a rich feature representational. Its convolutional layers have a huge receptive field, obtaining highly contextual details. The features of VGG 16 are robust to changes in scale, illumination, and rotation. The features of VGG 16 are transferable to distinct data sources and operations. Therefore, the VGG16 technique is chosen for the feature extraction task. VGG16 has three RGB channels. The VGG16 input tensor size is set to $244 \times 244$. Prioritized convolution layers are the VGG16's most distinguishing design parameters with size $3 \times 3$. The size of the max pool value is $2 \times 2$. The $1000$ channels are present in the final layer. The fully-connected layers of the first layer contain $4096$ channels and finally, the soft-max layer is set to the final layer.

Although the VGG 16 model is relatively effective in feature extraction, this model requires careful weight optimization for providing better feature representations. Moreover, poor weight optimization can result in overfitting issues. Therefore, an effective weight optimization is required for the VGG 16 model for improving the feature extraction task. For this, the RPAOSM-ESO-BCDNet algorithm is applied. This algorithm is suitable for selecting the optimal weights by utilizing the merits of two powerful algorithms such as AOSMA and ESOA. This algorithm also attained high convergence rates thus choosing the optimal weights quickly and properly. Therefore, the overfitting issue is minimized and the demand for computational resources is minimized. Finally, the RPAOSM-ESO-BCDNet highly increases the rate of accuracy. The objective function for this task is calculated by Eq. (1).

1 $$Oj_{1} = \mathop {\arg \,\min }\limits_{{\left\{ {L_{VGG16}^{weight} } \right\}}} \left( \frac{1}{acc} \right)$$

Here, the optimized weight of VGG-16 is $L_{VGG16}^{weight}$. The weight is optimized and selected in the interval of $\left[ {0,\,1} \right]$. The maximization of accuracy is measured using Eq. (2).

2 $$acc = \frac{{\left( {MO_{v} + TK_{y} } \right)}}{{\left( {MO_{v} + TK_{y} + MO_{n} + TK_{h} } \right)}}$$

**Fig. 3:**
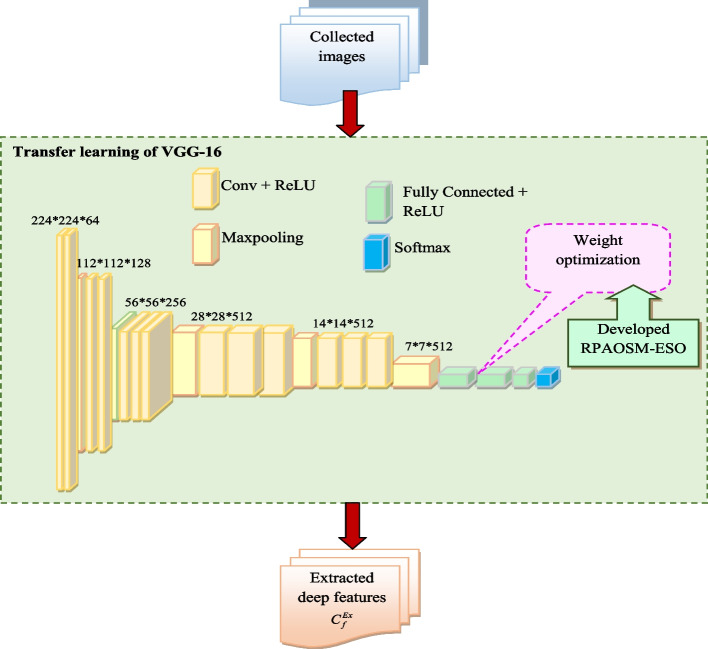
Structural representation of transfer learning of VGG-16-related feature extraction

The terms $MO_{v}$ and $MO_{n}$ are denoted as true positive and negative values. The terms $TK_{y}$ and $TK_{h}$ are indicated as false negative and positive values. Finally, the deep extracted features are retrieved and denoted by $C_{f}^{Ex}$. The structural representation of transfer learning of VGG-16-based deep feature extraction is shown in Fig. 3.

### AHDNAM-based breast cancer classification

The deep features $C_{f}^{Ex}$ are extracted and it is given to the AHDNAM-based classification section. Here, the DTCN and 1DCNN networks are integrated to form the hybridized network, and ASPP with attention mechanism is included in the model.

#### DTCN [30]

The deep extracted features are given to the DTCN classification section. It makes it possible for the DTCN network to generate precise data representations by using common dilated convolution. The input is utilized in both dilated convolutions and regular areas. The process of dilated convolution is measured using Eq. (3).

3 $$J\left( w \right) = \left( {b *_{h} j} \right)\left( w \right) = \sum\limits_{m = 0}^{o - 1} {j\left( m \right)} .b_{w - h.m}$$

Here, the parameter $J\left( w \right)$ denoted as the dilated parameter. It is added to the dilation layer. The term $I$ is the process of data transformation. The value $s$ is measured using Eq. (4).

4 $$r = Cev\left( {A + I\left( A \right)} \right)$$

DTCN includes the filter size, depth, and dilation factor. Finally, the residual connection lowers the gradient explosion and DTCN disappearance.

#### 1DCNN [31]

The deep extracted features are classified using the 1DCNN [31]. It enhances the implementation of the model. In 1-DCNN, 1D convolutions and sub-sampling are employed. The value $d_{n}^{p}$ is the computational loss and it is calculated by Eq. (5).

5 $$d_{n}^{p} = j\left( {\sum\limits_{m \in \,Qm} {d_{m}^{p - 1} } * O_{mn}^{p} + f_{n}^{p} } \right)$$

The term $p_{n}$ is the initial layer. The term $p$ is the common 1-DCNN network layer. The term $W_{n}^{p}$ is the last layer and it is calculated using Eq. (6).

6 $$W_{n}^{p} = N\left( {e_{m}^{p} } \right),\,\,m \in \,V_{n}^{p}$$

Here, the term $V_{n}^{p}$ is the pooling domain. The term $S$ is used to determine the 1-DCNN’s weight and biases. The term $S$ is measured using Eq. (7).

7 $$S = j\left( {f_{s} + x_{s} j_{z} } \right)$$

The feature in the pooling domain is indicated by $e_{m}^{p}$. The pooling layer kernel size is set to $w \times 1$.

#### ASPP [32]

To achieve the given feature map’s contextual multi-scale details [33], numerous convolutions with distinct expansion coefficientsareutilized with the goal of attaining multi-scale feature maps. For minimizing the changes in mapping feature size, the mapping features are developed by splicing by utilizing the SPP. Moreover, the depth-wise separable convolution is normally utilized when executing the input image’s channels. Hence, a new ASPP is implemented by integrating the depth-wise separable convolution [34] and the spatial pyramid pooling for separating the given image channel from spatial details.The ASPP enhances the view field for retrieving the multi-scale features. In the ASPP module, the gaps between the filter kernels and atrous convolution capture bigger feature maps. The feature maps are processed for each parallel branch in ASPP and it is used in a convolution, ReLU activation function, and batch normalization. Standard convolution is similar to atrous convolution, except atrous convolution's kernel is rarely used by adding zero rows and columns of weights. The dilation rate is noted by $t$. The atrous convolution output is measured using Eq. (8).

8 $$A\left[ o \right] = \sum\limits_{e = 1}^{e} {y\left[ {o + t.l} \right]} \,g\left[ e \right]$$

Here, the term is specified by $o$. The term $g$ is the weight and the location is represented by $l$. The ASPP module is used in the DTCN architecture.

#### Attention mechanism [35]

The attention method can increase the functionality of the neural networks by supporting them in concentrating on the related features of the given sequences. The attention method increases the accuracy of the classification process by contrasting the highly relevant image features. Also, the attention mechanism increases the efficiency and interpretability of the network.The attention mechanism is trained through back-propagation in the usual system. The attention method results in a weighted total at each location. The weights for each position are typically provided via the softmax layer, a deterministic approach with differentiable attention. Backpropagation is utilized in conjunction with the other elements of the network to do training since the complete process is described by a unique function. The detection of classified images is more closely related to the actual detection when using the attention technique. The result of the final outcome is a weight method. The AM value is measured by Eq. (9).

9 $$h_{l,w} = j\left( {Zk_{w} f} \right)$$

Here, the AM score is noted by $h_{l,w}$. The term $l$ indicates the soft-max layer. The term $d_{l,z}$ is calculated using Eq. (10).

10 $$d_{l,z} = \frac{{\exp \left( {h_{k,z} } \right)}}{{\sum\nolimits_{N = 1}^{W} {\exp \left( {h_{l,n} } \right)} }}$$

Here, the AM’s weight probability is represented by $d_{l,z}$.

#### AHDNAM

The extracted deep features are given to the developed AHDNAM-based breast cancer classification phase, denoted by $C_{f}^{Ex}$. It is used to accurately classify benign or malignant tumors using parameter optimization. By utilizing the mentioned DTCN, 1DCNN, ASPP, and the attention methods, the AHDNAM technique is developed. The DTCN offers more flexibility and quickly classifies the images. The 1DCNN technique is easy to train and has lower computational complexity. Therefore, these two techniques are selected for classifying breast cancer. However, when the input features are more, the DTCN model can't capture the complex features from the input features and also, the 1DCNN technique struggles to recognize the highly related features. In order to prevent these problems in these models, the ASPP is incorporated with the DTCN, and the attention method is incorporated with the 1DCNN model. Thus, the model becomes relatively effective. Although the AHDNAM model efficiently classifies breast cancer, the hyper-parameter utilization of DTCN and 1DCNN models is high. This minimizes the model's efficiency and also produces computational burdens. Therefore, tuning these model’s hyperparameters is necessary. For this, the developed RPAOSM-ESO is employed, which optimizes the parameters such as steps per epochs and hidden neurons from both DTCN and 1DCNN models in the AHDNAM model for increasing the accuracy and MCC and minimizing the FNR value. Thus the model performance is increased and the computational burdens are minimized by the designed RPAOSM-ESO algorithm in the classification process. Thus, the RPAOSM-ESO-BCDNet-based breast cancer diagnosis model is implemented for classifying breast cancer as benign and malignant. Initially, the extracted features $C_{f}^{Ex}$ are subjected to bothASPP-based optimized DTCN and attention-based optimized 1DCNN models. These two techniques produce the predicted scores and then these scores are averaged for producing the classified outcome for breast cancer. The objective function of this operation is given in Eq. (11).

11 $$Oj_{2} = \mathop {\arg \,\min }\limits_{{\left\{ \begin{subarray}{l} T_{DTCN}^{Hidden} ,K_{DTCN}^{Epoch} \\ P_{1DCNN}^{Hidden} ,M_{1DCNN}^{Epoch} \end{subarray} \right\}}} \left( {\frac{1}{acc} + \frac{1}{MCC} + FNR} \right)$$

In the DTCN, the optimized hidden neuron count is noted by $T_{DTCN}^{Hidden}$ and it is chosen in the range of $\left[ {5,\,255} \right]$. The steps per epoch are optimized $K_{DTCN}^{Epoch}$ and it is taken in the interval of $\left[ {10,\,50} \right]$. In the 1DCNN, the hidden neuron count is optimized $P_{1DCNN}^{Hidden}$ and it is selected in the interval of $\left[ {5,\,255} \right]$. The steps per epoch are optimized $M_{1DCNN}^{Epoch}$ and it is chosen in the interval of $\left[ {10,\,50} \right]$. The MCC parameter is validated using Eq. (12).

12 $$MCC = \frac{{TK_{y} \times TK_{h} - TK_{y} \times MO_{v} }}{{\sqrt {\left( {TK_{y} + MO_{n} } \right)\left( {TK_{y} + MO_{v} } \right)\left( {TK_{h} + TK_{y} } \right)} \left( {TK_{h} + MO_{v} } \right)}}$$

The MCC outcomes are more reliable outcomes based on the training. The formula of FNR is given in Eq. (13).

13 $$FNR = \frac{{TK_{y} }}{{TK_{y} + MO_{n} }}$$

**Fig. 4:**
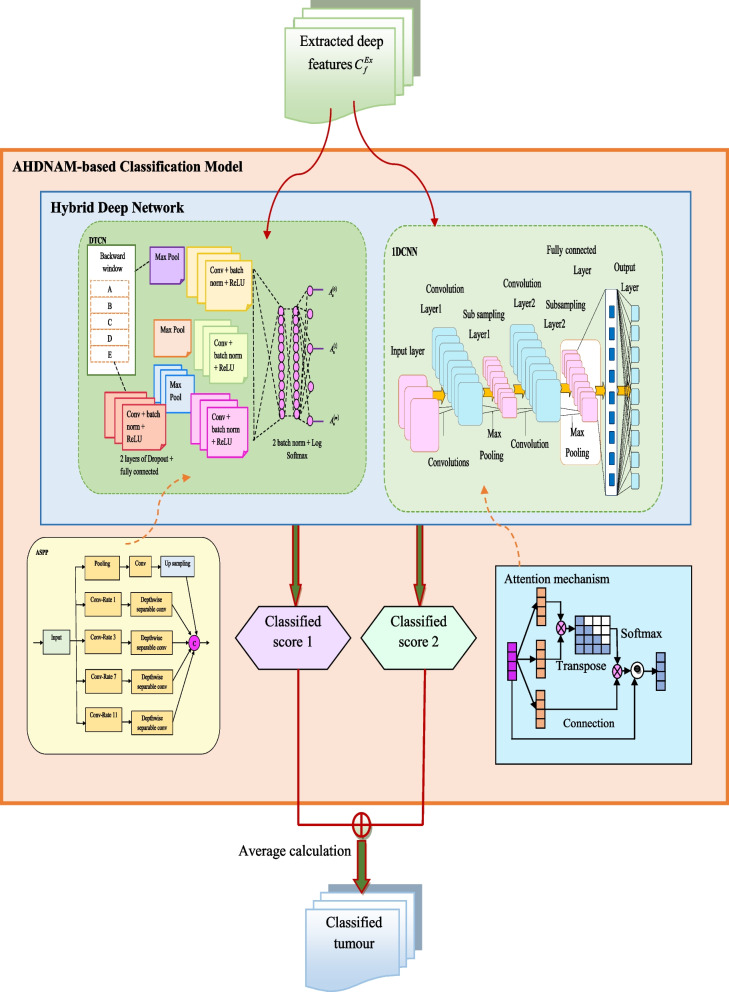
Structural representation of AHDNAM-based breast cancer classification model

The FNR is the false negative ratio with total positive. The structural representation of the AHDNAM-based breast cancer classification system is displayed in Fig. 4.

## Overall process in BCDNet model using deep networks with heuristic-based optimal tuning of parameters

### Formulation of BCDNet for breast cancer diagnosis

**Fig. 5:**
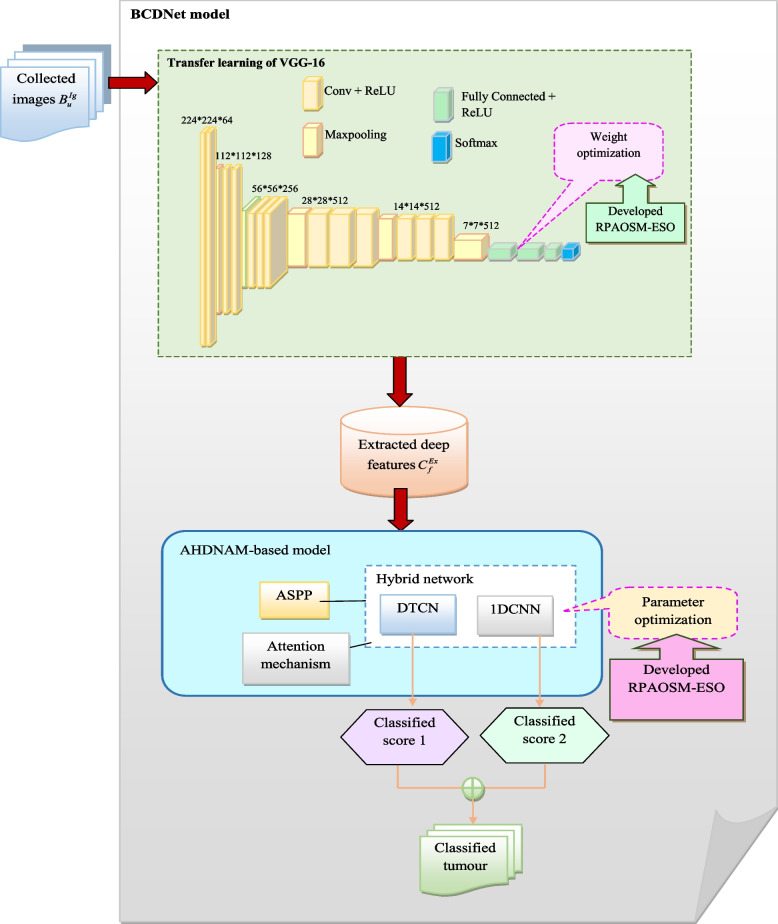
Structural representation of developed BCDNet for diagnosing breast cancer

An effective BCDNet is introduced in this research work for classifying breast cancer as benign and malignant. This designed automated model helps medical practitioners provide better treatment recommendations and also helps to minimize mortality rates. The designed BCDNet utilizes deep learning thus increasing the classification process’s effectiveness than the previous models. The BCDNet model is performed in two stages: feature extraction and classification. At first, from the standard resource, the ultrasound images $B_{u}^{Ig}$ are garnered and utilized as input for the suggested work. Since the existing imaging tools such as MRI and mammography are highly expensive, ultrasound images are highly preferred for the classification task, which is non-invasive and low cost. Further, the gathered ultrasound images $B_{u}^{Ig}$ are fed into the BCDNet model. In the first stage, the extraction of features is carried out for obtaining prominent features for the classification task. This feature retrieval process can minimize the computational time and also help the classification process by providing the related features. For this purpose, the transfer learning of VGG-16 is used to extract the deep features.This technique utilizes the knowledge from the pre-trained techniques and helps to minimize the dimensionality problems. In this, the corresponding weights network weights are determined optimally by the RPAOSM-ESO. Thus, the dice coefficient and accuracy of this phase are increased. This algorithm is a hybrid algorithm designed by including the traditional AOSMA and ESOA with an adaptive mechanism for increasing the performance of the classification and feature extraction process. The obtained deep features are given to the classification phase, and it is denoted by $C_{f}^{Ex}$. The classification is performed using the AHDNAM model.The AHDNAM is a hybrid network, which includes diverse powerful strategies for making the classification more effective. The proposed technique is relatively different from the conventional deep learning models, which don’t perform parameter tuning and also having the demand more processing time. Also, the existing deep models always struggle to capture the related and rich features for the classification tasks. But, the developed model employs the ASPP and attention methods that help to choose the complex and related features for the classification process, thus the accuracy and the efficiency of the model are increased. The model included the DTCN, 1DCNN, ASPP, and the attention models. Here, the ASPP is incorporated with the DTCN, while the attention method is incorporated with the 1DCNN technique. This hybrid network efficiently performs the classification process and produces the predicted outcomes as 1 and 2. These two predicted solutions are then averaged for achieving the final classification outcome. Here, the DTCN, and 1DCNN technique’s parameters are determined optimally by the recommended RPAOSM-ESO. This increased the accuracy and MCC values and at the same time, it helped to minimize the FNR values. Thus, the AHDNAM-based classification model is used to effectively classify the tumor. The structural diagram of the breast cancer diagnosis system using deep learning is shown in Fig. 5.

### Implemented RPAOSM-ESO

By revising the traditional AOSMA and ESOA, the RPAOSM-ESO is designed in this work.

#### Purpose

The designed RPAOSM-ESO algorithm is a hybrid algorithm, which is used to enhance the performance of feature extraction with weight optimization for transfer learning of the VGG-16 method and classification with parameter optimization for the AHDNAM method. The designed RPAOSM-ESO algorithm is employed to optimize the weights for increasing the accuracy and dice coefficient in the feature extraction. Also, it is employed for optimizing the parameters like epochs and hidden neuron count of the 1DCNN and DTCN techniques for improving the accuracy and MCC and minimizing the FNR value in the classification.

#### Novelty

The developed RPAOSM-ESO is developed by integrating the algorithms such as existing AOSMA and ESOA. The AOSMA is utilized for constructing the RPAOSM-ESO. The AOSMA provides greater convergence and diversification rates. Moreover, this algorithm can properly choose the solutions for difficult optimization issues. On the other hand, ESOA optimization contains strong searching capabilities. Due to its simple structure, it effectively escapes from the local optimum issue and also balances the exploitation and exploration phases. Therefore, traditional algorithms such as AOSMA and ESOA are chosen for this research work. However, AOSMA is stuck between local optimum and also it gives an imbalance function of exploitation and exploration phase. The ESOAalso faces some issues such as poor convergence rates. To resolve these problems in both AOSMA and ESOA, these two algorithms are combined with an adaptive method. This combined algorithm is named RPAOSM-ESO. The new random number is indicated by $RR$. In the conventional strategies, the random parameter is linearly increased using the range of $\left[ {0,1} \right]$.This may cause more problems as mentioned before due to its uncertainty. So, refining this random integer can prevent these problems in the existing algorithms.In the adaptive concept of RPAOSM-ESO, a new random integer $RR$ is defined by considering the current and maximum iteration values and it is given in Eq. (14).

14 $$RR = - u * \left( {\frac{{\left( { - 1} \right)}}{Max\_Iter}} \right)$$

Here, the parameter $Max\_Iter$ is the maximum value of iterations in the developed RPAOSM-ESO algorithm. The present iteration is represented by $u$. Thus, this new random integer is developed. The working functionality of the RPAOSM-ESO is explained as follows. If the designed random integer $RR$ is divisible by 0.02 (i.e.$RR\% 0.02 = = 0$,), then the AOSMA is applied for updating the positions. Or else (i.e.$RR\% 0.02 \ne 0$,), the ESOA is applied for updating the positions. Here, the newly updated random parameter is used to enhance the effectiveness of the process. The existing AOSMA and ESOA are explained below.

#### AOSMA [36]

The AOSMA has gained popularity in the field of optimizationwith the help of successfully combining exploration and exploitation to arrive at an ideal or nearly ideal solution. The SMA's exploitation and exploration are constrained. The steps of the AOSMA are provided below.

1. In the beginning, the population of the slime mould is initialized in a random way and its fitness function calculation is carried out.
2. Further, the updating of local fitness and global fitness values is carried out on the basis of the global best solution space.
3. An adaptive decision strategy is then carried out by opposition-based learning.
4. The best positions are stored and in the end, the global best solution is returned as outcome.

#### ESOA [36]

The great egret, middle egret, little egret, and yellow-billed egret are four bird species together known as egrets, and they are all distinguished by their stunning white plumage. These behaviors are used to implement the ESOA algorithm. The three strategies are used to enhance the ESOA. That is wait and sit, aggressive,and the discriminate condition strategy. The ESOA’s steps are provided as follows.

1. The egret’s starting populations are initialized in a random way and the objective functions are calculated.

2. Based on the sit and wait mechanism, the position updating is carried out and the position updating on the basis of the aggressive mechanism is carried out.

3. Finally, the best positions are recorded and the optimal solutions are given as outcomes.

The pseudo-code of the suggested RPAOSM-ESO is given in Algorithm 1.

Algorithm 1. Implemented RPAOSM-ESO.
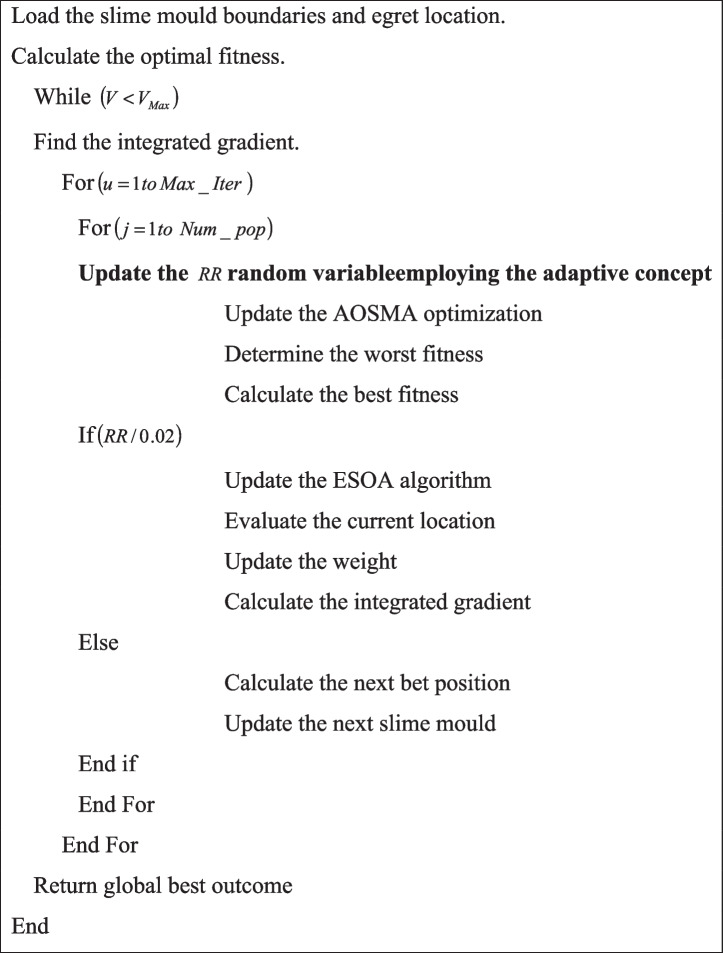


## Results and discussions

### Experimental setup

The developed breast cancer diagnosis system employing deep hybrid learning was implemented for predicting benign or malignant tumors using a Python environment. The developed breast cancer diagnosis system effectiveness was compared to traditional heuristic algorithms and techniques. A chromosome length of 6, a maximum iteration of 50, and a population size of 10 were employed for the investigational analysis. The RPAOSM-ESO utilized two parameters such as beta1 = 0.9, and beta2 = 0.99. The existing Mine Blast Optimization (MBO) [37], Black Widow Optimization (BWO) [38], AOSMA [36], and ESOA [39] algorithms were utilized for analyzing the implemented model. These algorithms have been selected in this experiment since these models are recent and effective. Moreover, conventional approaches like DTCN [30], 1DCNN [31], MobileNet [40], and ASSP-DTC-1DCNN-AM [32] were used for the experimental evaluation.The experimental environment of the developed model is explained as follows. The programming language of the developed model was Python and the software was Pycharm with version 3.11 and Anaconda with version 3. The system OS was Windows with 8 GB RAM and 500 GB ROM. The parameters such as hidden neuron count and the epochs in the 1DCNN and DTCN are selected in the range of [5, 255], and [5–50] respectively.

### Evaluation measures

The offered breast cancer diagnosis system employing deep hybrid learning included some performance metrics and it is given below.

- NPV:$$npv = \frac{{MO_{v} }}{{MO_{v} + TK_{y} }}$$
- F1-score: $$f1 = \frac{{2 \times MO_{n} }}{{2MO_{n} + TK_{h} + TK_{y} }}$$
- FPR: $$fpr = \frac{{MO_{v} }}{{MO_{v} + TK_{h} }}$$
- Specificity: $$sy = \frac{{TK_{h} }}{{TK_{h} + MO_{n} }}$$
- FDR: $$fdr = \frac{{TK_{h} }}{{MO_{v} + TK_{y} }}$$
- Precision: $$pr = \frac{{TK_{h} }}{{TK_{y} + MO_{v} }}$$
- Sensitivity: $$sen = \frac{{MO_{n} }}{{MO_{n} + TK_{h} }}$$

### Accuracy analysis of developed breast cancer diagnosis model

**Fig. 6:**
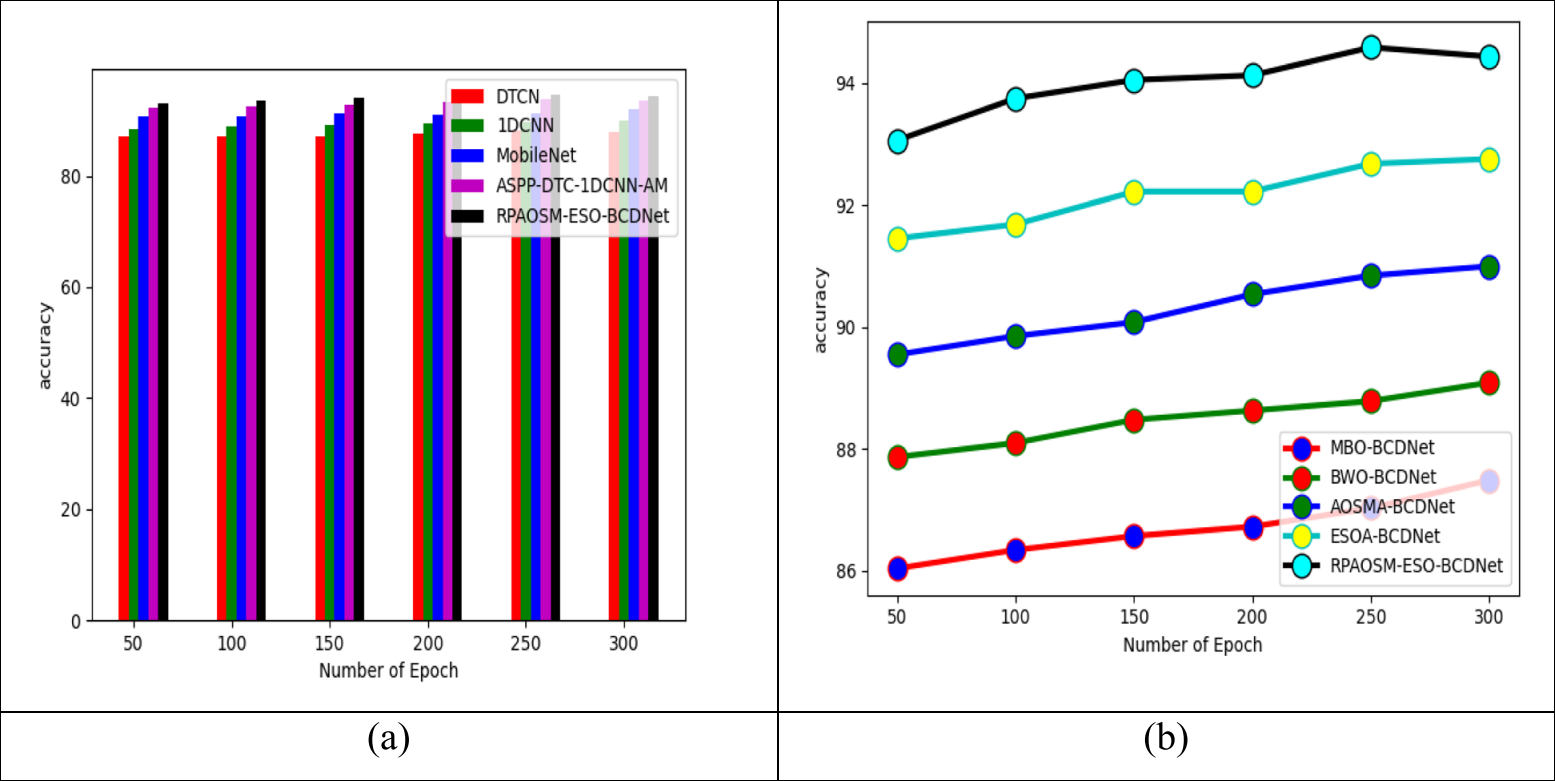
Accuracy analysis of developed diagnosis of breast cancer system with respect to “(**a**) Existing classifiers, and (**b**) Existing algorithms”

The effectiveness of the implemented breast cancer diagnosis model using hybrid deep learning is compared in terms of accuracy with existing methods and heuristic algorithms and it was depicted in Fig. 6.The accuracy-based examination is utilized to analyze how effectively the developed RPAOSM-ESO-BCDNet model classifies the breast cancer. The number of epochs is utilized to assess the accuracy of the model since the number of epochs can determine the model performance precisely. The RPAOSM-ESO-BCDNet-based breast cancer diagnosis model’s accuracy is highly increased by 1.13% ofDTCN, 2.29% of1DCNN, 3.48% of MobileNet, and 4.70% ofASSP-DTC-1DCNN-AM when the number of epochs is 150. Thus, the RPAOSM-ESO-BCDNet-based breast cancer diagnosis model effectiveness is assessed with traditional algorithms and techniques and ensures its effectiveness and supremacy over other models.

### Performance analysis of developed breast cancer diagnosis model

**Fig. 7:**
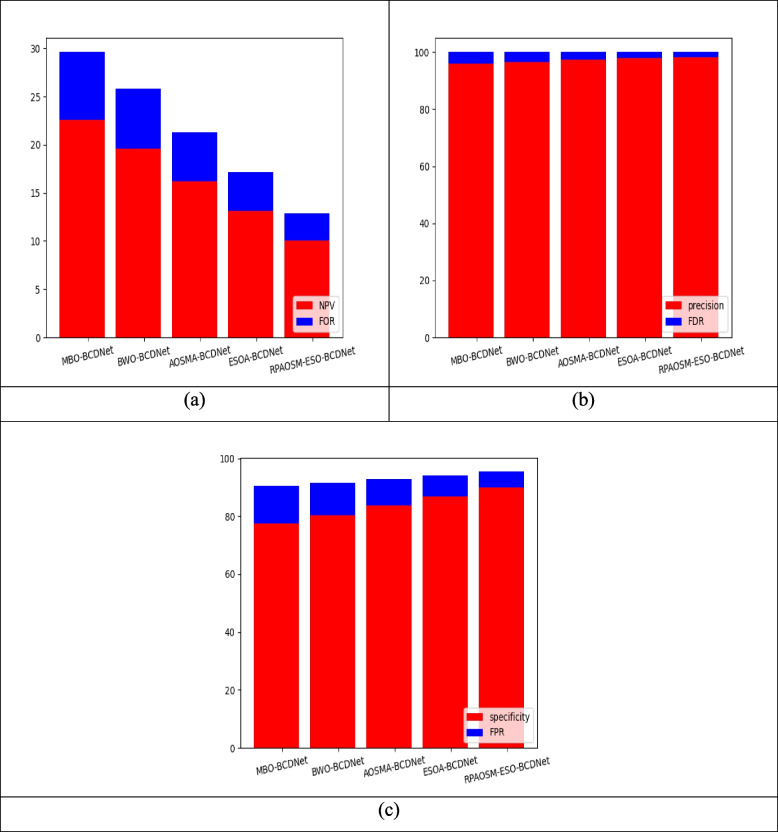
Performance evaluation on developed breast cancer diagnosis system over several algorithms with respect to “(**a**) NPV vs FOR, (**b**) Precision vs FDR, and (**c**) Specificity vs FPR”

**Fig. 8:**
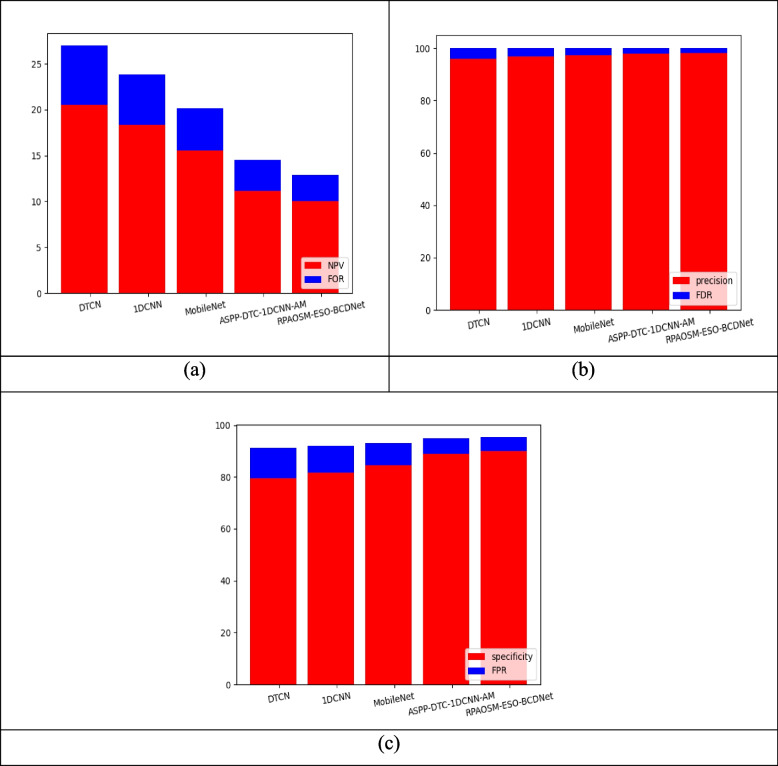
Performance analysis on developed breast cancer diagnosis system over several methods with respect to “(**a**) NPV vs FOR, (**b**) Precision vs FDR, and (**c**) Specificity vs FPR”

Figures 7 and 8 show the performance comparison of theimplemented breast cancer diagnosis model over various algorithms and conventional approaches. Based on distinct performance metrics, this experiment is carried out. The NPV of the RPAOSM-ESO-BCDNet-based breast cancer diagnosis model is increased by 20.3% of MBO-BCDNet, 19.7% of BWO-BCDNet, 16% of AOSMA-BCDNet, and 11% of ESOA-BCDNet. For the experimental analysis, the RPAOSM-ESO-BCDNet-based implemented breast cancer diagnosis model provided a better NPV value. Hence, the effectiveness of the developed breast cancer diagnosis process is proved with distinct performance metrics over traditional models and algorithms. The experiment proves that the RPAOSM-ESO-BCDNet can recognize breast cancer in early times with low error rates thus improving the life quality of the model.

### Cost function validation of developed RPAOSM-ESO

**Fig. 9:**
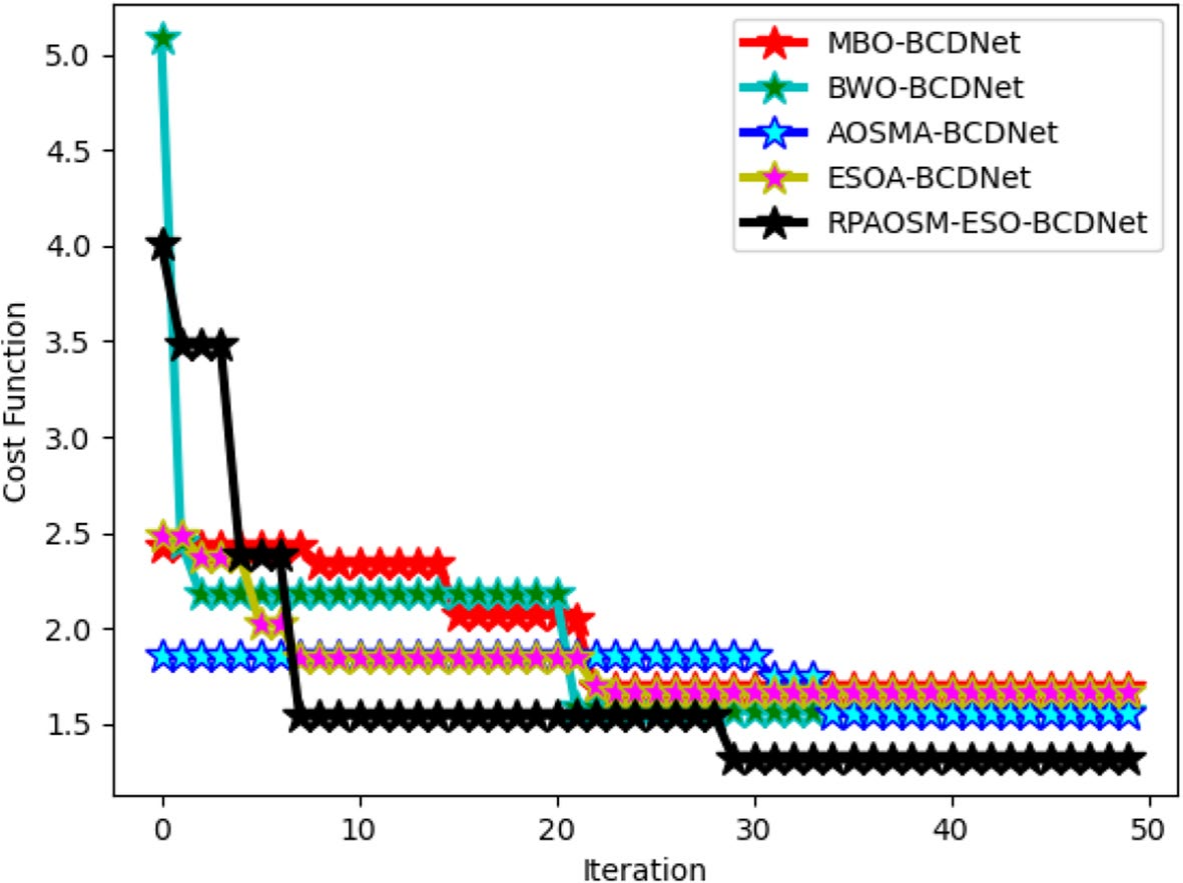
Cost function evaluation on developed RPAOSM-ESO algorithm for implemented classification process of breast cancer over different heuristic strategies

The RPAOSM-ESO algorithm’s cost function is assessed by varying the iteration values over various heuristic strategies as shown in Fig. 9. The RPAOSM-ESO’scost function is reduced by 25% of MBO, 28.5% ofBWO, 37.5% of AOSMA, and 34.7% of ESOA at the 20th iteration. Compared to conventional algorithms, the RPAOSM-ESO-BCDNet-based breast cancer diagnosis model obtained a low-cost function. This analysis proved that the suggested RPAOSM-ESO algorithm can efficiently perform the parameter and weight optimization than the other heuristic models thus providing greater performance for the BCDNet-based breast cancer diagnosis. This also proves that the RPAOSM-ESO algorithm has higher convergence than the previous algorithms. Moreover, because of this RPAOSM-ESO algorithm, the accuracy of the classification process is relatively increased.

### ROC evaluation of developed breast cancer detection system

**Fig. 10:**
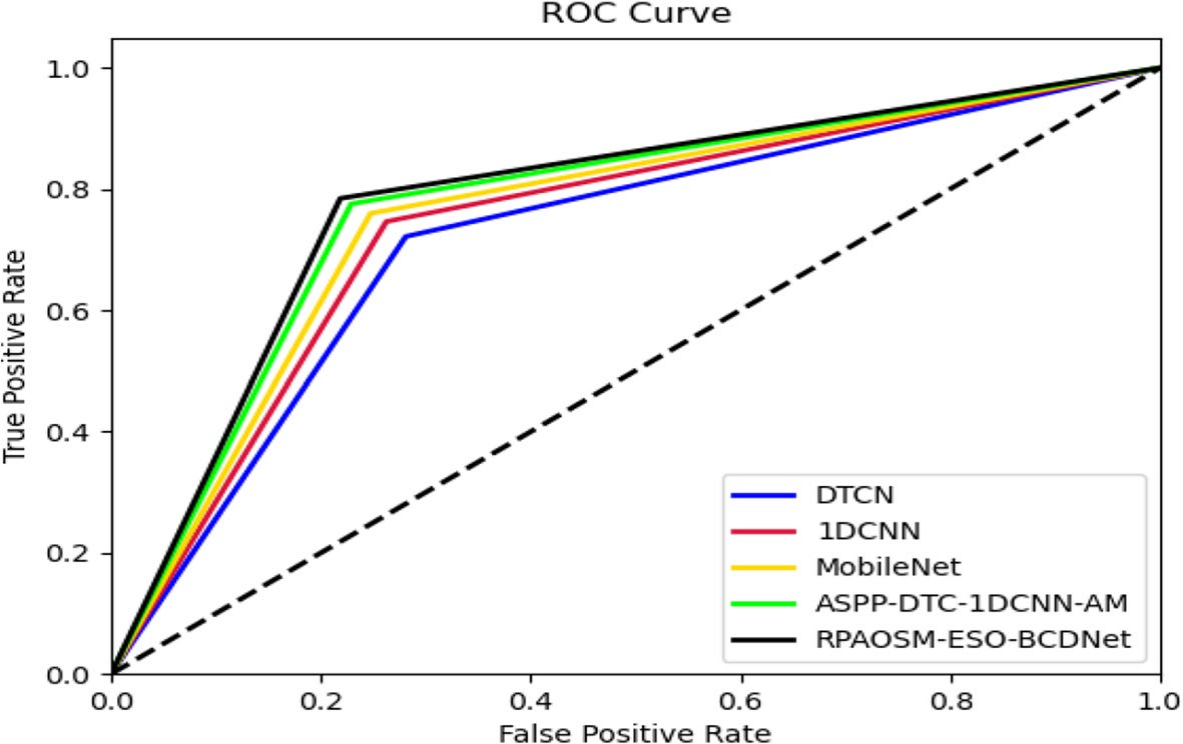
ROC analysis of developed diagnosis systemover different classifiers

The effectiveness of the developed BCDNet is analyzed utilizing ROC over distinct classifiers and depicted in Fig. 10. The false and true positive rates are varied for this experiment. The RPAOSM-ESO-BCDNet-based breast cancer diagnosis model’s effectiveness is enhanced by 14.2% of MBO, 6.6% of BWO, 15.9% of AOSMA, 31.14% of ESOA. Thus, the RPAOSM-ESO-BCDNet-based breast cancer diagnosis system showed greater performance in terms of ROC analysis than previous systems. This analysis also elucidated that the error values of the suggested BCDNet are highly reduced in the diagnosis process than the conventional techniques thus helping the accurate treatment recommendations.

### Confusion matrix of developed diagnosis model

**Fig. 11:**
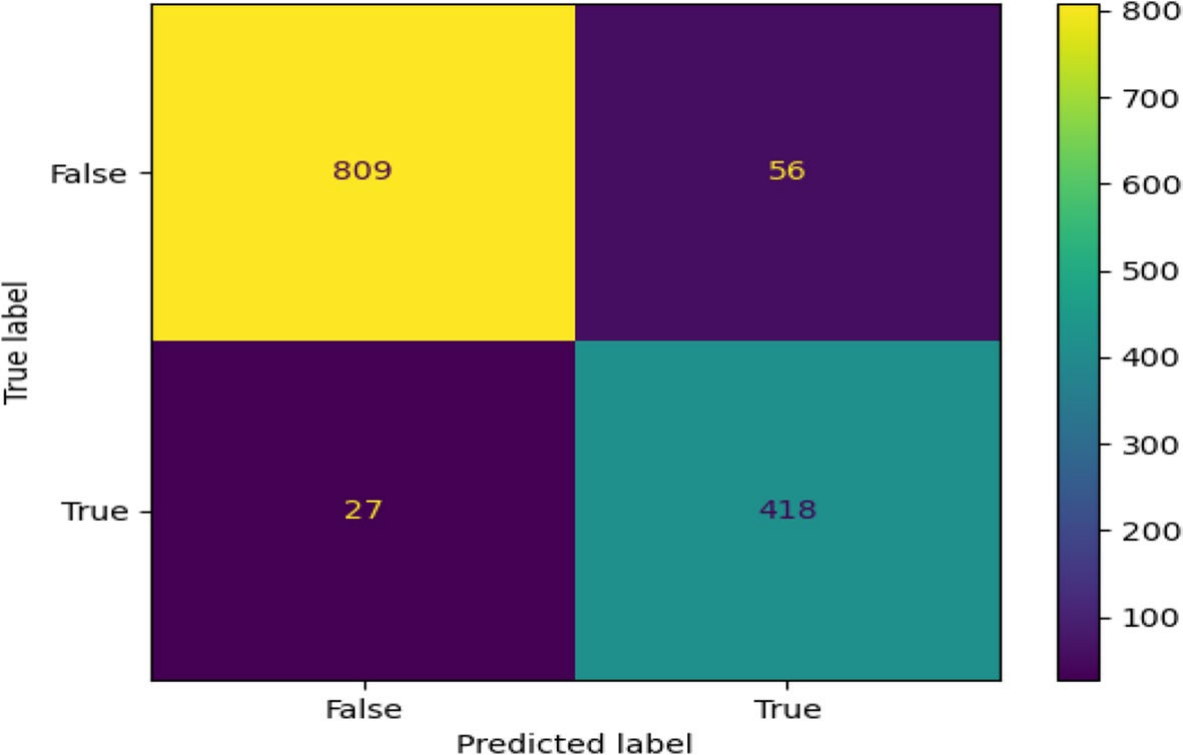
Confusion matrix analysis on designed breast cancer diagnosissystem using deep hybrid learning

The performance of the implemented diagnosis of breast cancer model is analyzed and shown in Fig. 11 in terms of the confusion matrix. By considering the predicted and true labels, the accuracy of the model is examined. The confusion matrix showed a high accuracy value thus provingthe effectiveness of the system. Thus, the RPAOSM-ESO-BCDNet-based breast cancer diagnosis model performance provided low error values thus improving the classification process of breast cancer than the previous models.

### Overall analysis of the developed system

Comparison of the suggested breast cancer diagnosis modelover heuristic algorithms is shown in Table 2 and the comparison of the suggested model over traditional methods is shown in Table 3. The RPAOSM-ESO-BCDNet-based breast cancer diagnosis model’s FNR is decreased by 1.86% ofMBO, 3.9% ofBWO, 6.37% ofAOSMA, and 8.77% ofESOA.Thus, the developed RPAOSM-ESO-BCDNet produced lower false rates than the existing models. Moreover, the accuracy of the suggested RPAOSM-ESO-BCDNet is increased by 67% of DTCN, 51.7% of 1DCNN, 33.9% of MobileNet, and 8.1% of ASPP-DTC-1DCNN-AM. Hence, the superiority of the RPAOSM-ESO-BCDNet-based diagnosis model is ensured and guaranteed that the model can help the early diagnosis of breast cancer than the existing models.

**Table 2:** Performance validation of the developed system over different algorithms

Terms	MBO-BCDNet [37]	BWO-BCDNet [38]	AOSMA-BCDNet [36]	ESOA-BCDNet [39]	RPAOSM-ESO-BCDNet
Accuracy	87.0229	88.77863	90.83969	92.67176	94.58015
Sensitivity	87.19101	88.53933	90.5618	92.35955	94.60674
FNR	86.93642	88.90173	90.98266	92.83237	94.56647
Specificity	77.44511	80.40816	83.78378	86.89218	89.95726
FPR	13.06358	11.09827	9.017341	7.16763	5.433526
Precision	12.80899	11.46067	9.438202	7.640449	5.393258
FDR	86.93642	88.90173	90.98266	92.83237	94.56647
NPV	22.55489	19.59184	16.21622	13.10782	10.04274
FOR	7.045735	6.219512	5.066345	4.062127	2.850356
F1_Score	82.0296	84.27807	87.04104	89.54248	92.22344
MCC	0.722394	0.757974	0.801185	0.840027	0.88134

**Table 3:** Performance validation of diagnosis system over different methods

Terms	DTCN [30]	1DCNN [31]	MoibleNet [40]	ASPP-DTC-1DCNN-AM [32]	RPAOSM-ESO-BCDNet
Accuracy	88.24427	89.69466	91.37405	93.81679	94.58015
Sensitivity	88.08989	89.88764	91.46067	93.48315	94.60674
FNR	88.3237	89.59538	91.32948	93.98844	94.56647
Specificity	79.51318	81.63265	84.43983	88.88889	89.95726
FPR	11.6763	10.40462	8.67052	6.011561	5.433526
Precision	11.91011	10.11236	8.539326	6.516854	5.393258
FDR	88.3237	89.59538	91.32948	93.98844	94.56647
NPV	20.48682	18.36735	15.56017	11.11111	10.04274
FOR	6.487148	5.487805	4.589372	3.444181	2.850356
F1_Score	83.58209	85.5615	87.81014	91.12815	92.22344
MCC	0.747006	0.77796	0.81307	0.864522	0.88134

### Statistical analysis of the RPAOSM-ESO

Table 4 displays the statistical analysis of RPAOSM-ESO over other existing heuristic strategies. For analyzing the performance of the RPAOSM-ESO, the statistical measures including “best, worst, mean, median, and the standard deviation” are supported. The RPAOSM-ESO’s performance is increased by 8.07% ofMBO, 4.99% ofBWO, 11.9% ofAOSMA, and 14.6% ofESOAwhen considering the mean value.Thus, this investigation shows that the RPAOSM-ESOattained better performance rates for the optimization approach for the BCDNet than the existing algorithms. Moreover, it has been confirmed that the incorporation of RPAOSM-ESO in the BCDNet increased the accuracy rates than the conventional algorithms.

**Table 4:** Statistical evaluation of implemented RPAOSM-ESO over different algorithms

Terms	MBO- [37]	BWO-BCDNet [38]	AOSMA-BCDNet [36]	ESOA-BCDNet [39]	RPAOSM-ESO-BCDNet
BEST	2.423906	5.082712	1.854273	2.48182	4.008066
WORST	1.683622	1.566266	1.55242	1.666036	1.312354
MEAN	1.948037	1.888587	1.750246	1.808994	1.66286
MEDIAN	1.683622	1.583892	1.851872	1.666036	1.544823
STD	0.314948	0.547858	0.13816	0.225161	0.630674

#### Robustness analysis of the developed system

**Fig. 12:**
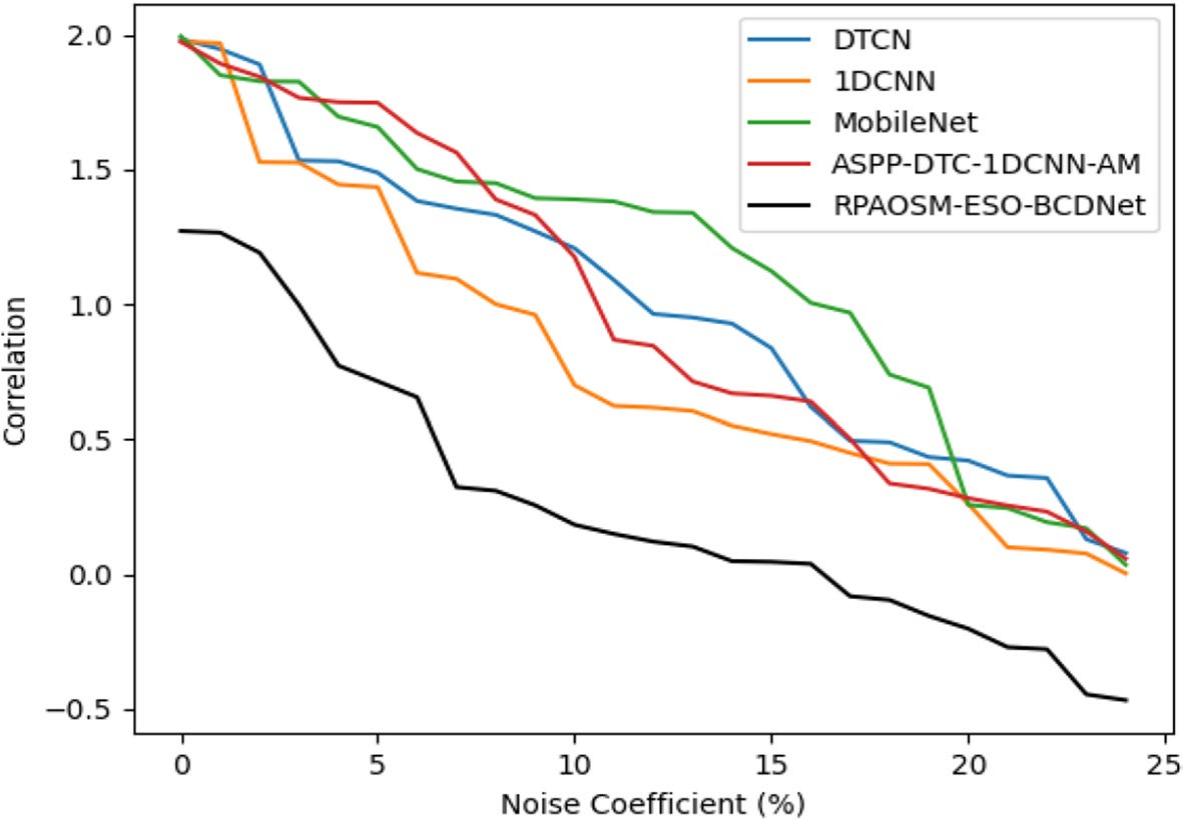
Robustness analysis of the designed system

The robustness analysis of the designed model has been provided in Fig. 12. The robustness examination supports evaluating how well a developed model works under distinct situations, or how to manage flexibility in an unknown situation. By correlating the noise coefficient, the suggested framework’s robustness is investigated. When the noise coefficient is 15, the suggested framework’s correlation is reduced by 34.55% of DTCN, 32.33% of 1DCNN, 35.66% of MobileNet, and 39% of ASPP-DTC-1DCNN-AM respectively. Hence, the experiment ensured that the suggested RPAOSM-ESO-BCDNet obtained relatively higher robustness than the previous models thus ensuring its effectiveness in distinct and unknown situations.

#### Ablation experiment of the developed system

The ablation study experiment for the implemented classification model is presented in Table 5. This experiment has helped to investigate the accuracy of the developed RPAOSM-ESO-BCDNet model with other deep learning models. The accuracy of the RPAOSM-ESO-BCDNet is 94.5%. Hence, this study ensures that the implemented RPAOSM-ESO-BCDNet model can produce highly accurate solutions by incorporating the RPAOSM algorithm than the conventional models.

**Table 5:** Ablation study of implemented model

Models	Accuracy (%)
1DCNN [31]	90.6
DTCN [30]	91.4
1DCNN + DTCN [30, 31]	91.4
RPAOSM-1DCNN	93.2
RPAOSM-DTCN	93.6
RPAOSM-ESO-BCDNet	94.5

#### Computational efficiency analysis of the developed system

The computational efficiency of the implemented RPAOSM-ESO-BCDNet is analyzed in Table 6. In this, the implemented RPAOSM-ESO-BCDNet technique’s computational time is evaluated over traditional algorithms and techniques for proving the effectiveness of the RPAOSM-ESO-BCDNet. The experiment has shown that the RPAOSM-ESO-BCDNet attained 13 min for completing the process, which is highly lower than the conventional techniques. The computational efficiency of the designed RPAOSM-ESO-BCDNet is more and it is proved in this experiment.

**Table 6:** Computational efficiency analysis ofthe implemented model

Experiments based on Algorithms	
**Algorithms**	**Time (mins)**
MBO- [37]	12.74
BWO-BCDNet [38]	15.36
AOSMA-BCDNet [36]	13.749
ESOA-BCDNet [39]	13.802
RPAOSM-ESO-BCDNet	13.0683
**Experiments based on Classifiers**	
DTCN [30]	18.3
1DCNN [31]	16.3
MoibleNet [40]	14.6
ASPP-DTC-1DCNN-AM [32]	13.509
RPAOSM-ESO-BCDNet	13.0683

#### State-of-the-art analysis of the developed system

Table 7 provides the state-of-the-art analysis of the implemented model. The accuracy, sensitivity, and specificity of the developed RPAOSM-ESO-BCDNet are analyzed with the state-of-the-art models. The sensitivity of the implemented RPAOSM-ESO-BCDNet is increased by 5.05% of VGG-19, 6.38% of CRNN, 1.95% of MDA-Net, and 1.68% of DGANet accordingly. Hence, the experiment ensured that the developed RPAOSM-ESO-BCDNet provided more accurate classifications for breast cancer than the state-of-the-art models. Moreover, it has been confirmed that the hybridization of this technique can prevent misclassifications and manual intervention.

**Table 7:** State-of-the-art analysis of developed system

Terms	VGG-19 [18]	CRNN [19]	MDA-Net [21]	DGANet [25]	RPAOSM-ESO-BCDNet
Accuracy	84.2	84.52	87.68	88.48	90
Sensitivity	85.6	84.4	88.4	88.64	90.16
Specificity	82.8	84.64	86.96	88.32	89.84

## Conclusion

A new breast cancer diagnosis system has implemented in this research article for classifying breast cancer as benign and malignant. This diagnosis process utilized the transfer learning-based optimized deep model for the feature extraction and optimized hybrid deep learning mechanism for the classification. Thus, the model helped in the early diagnosis process and also helped to minimize the mortality rates. The summary of the implemented model was as follows. In the beginning, theultrasound images were collected from the dataset. The gathered images were given to the BCDNet model. The feature extraction was performed using transfer learning of the VGG-16 method.Here, the developed RPAOSM-ESO was used to optimize the weights of VGG-16 for maximizing accuracy during the extraction of the deep features. After that, the extracted deep features were fed into the classification. The extracted features were given in the classification section. The classification was performed using anAHDNAM technique, which includedASPP-based DTCN and attention-based 1DCNN. These hybrid techniques produced the predicted scores and then the scores were averaged for attaining the classified outcomes. Here, the implemented RPAOSM-ESO was used to optimize the parameters like hidden neurons and epochs of DTCN and 1DCNN thus minimizing the FNR value and effectively increasing the accuracy and MCC. At last, the developed model accurately classified the benign or malignant tumor. The RPAOSM-ESO-BCDNet-based breast cancer diagnosis model’s accuracy was increased by1.14% of DTCN, 3.21% of 1DCNN, 3.57% of MobileNet, and 5.9% of ASSP-DTC-1DCNN-AM.Thus, the comparison of the developed breast cancer diagnosis model over existing models showed superior performance rates than the conventional models.

### Theoretical and practical implications

The breast cancer diagnosis model has been implemented in this research work utilizing ultrasound images. The ultrasound imaging tool is non-invasive and low cost than the conventional imaging models. Therefore, this work utilized ultrasound images for the classification of breast cancer. Further, the transfer learning of VGG 16 is utilized in the work for retrieving the significant features. The VGG 16 model has more layers hence it can choose highly significant and related features than the pre-trained models and the training time of the model is also reduced because of this model. Here, the weights of VGG 16 are optimized by the hybrid algorithm. The extracted features are processed by the optimized hybrid deep learning. By averaging the predicted scores of the deep learning models, the classified outcomes are achieved as benign and malignant. Here also, the hybrid algorithm is used to optimize the deep model’s parameters. This optimized hybrid network not only increases the accuracy but also prevents misclassifications.

### Practical advantages

The designed network can support medical institutions, research centers, academia, hospitals, and so on. This developed model can help medical experts in minimizing their workloads and saving diagnosis time. Moreover, the misclassifications and the false alarms can be resolved by the suggested diagnosis model. Therefore, the lifespan of the subjects can be increased.

## Research limitations

Though the implemented diagnosis model of breast cancer is relatively accurate and effective, the suggested model didn’t perform the image pre-processing for reducing the noise in the original ultrasound images. This may affect the classification process’s outcomes. Moreover, the developed model utilized a single dataset and didn't focus on large-scale datasets. When processing large-scale data sources, the developed model can face the issues of overfitting, long training time, difficulty in handling the data diversity, and so on.

### Future research suggestions

In future work, the developed model will be improved by introducing an effective image pre-processing model for minimizing the noise in the raw ultrasound images, and also, the implemented model will be processed with distinct and large-scale datasets in future work. For improving the effectiveness of the cancer classification process, the developed model will be improved by an ensemble-based deep learning model.

## Data Availability

The ultrasound images are gathered from the online database "Kaggle" using the link”https://www.kaggle.com/datasets/aryashah2k/breast-ultrasound-images-dataset: Access date: 2023–06-07".

## Nomenclatures

AM: Attention Mechanism
AOSMA: Adaptive Opposition Slime Mould Algorithm
ASSP: Atrous Spatial Pyramid Pooling
BCDNet: Breast Cancer Diagnosis Network
CNN: Convolutional Neural Network
CRNN: Convolutional Recurrent Neural Network
DGANet: Dual Global Attention Neural Network
DTCN: Deep Temporal Context Networks
ESOA: Egret Swarm Optimization Algorithm
FDR: False Discovery Rate
FNR: False Negative Rate
FPR: False Positive Rate
MBO: Mine Blast Optimization
MCC: Matthews Correlation Coefficient
MDA-Net: Multiscale Dual Attention Network
NPV: Negative Predictive Value
RPAOSM-ESO: Random Parameterized Adaptive Opposition Slime Mould with Egret Swarm Optimization
U-Net: U-shaped Convolutional Neural Network
VGG16: Visual Geometry Group 16 Layers
1DCNN: One Dimensional Convolution Neural Networks
CAD: Computer-Aided Diagnosis
MRI: Magnetic Resonance Imaging
GA: Genetic Algorithm
PSO: Particle Swarm Optimization
